# Amplification of early drought responses caused by volatile cues emitted from neighboring tea plants

**DOI:** 10.1038/s41438-021-00704-x

**Published:** 2021-11-15

**Authors:** Jieyang Jin, Mingyue Zhao, Ting Gao, Tingting Jing, Na Zhang, Jingming Wang, Xianchen Zhang, Jin Huang, Wilfried Schwab, Chuankui Song

**Affiliations:** 1 State Key Laboratory of Tea Plant Biology and Utilization, International Joint Laboratory on Tea Chemistry and Health Effects, Anhui Agricultural University, 230036 Hefei, Anhui P. R. China; 2 Biotechnology Institute, Chengdu Newsun Crop Science Co., Ltd, 610212 Chengdu, P. R. China; 3 Biotechnology of Natural Products, Technische Universität München, Liesel-Beckmann-Str. 1, 85354 Freising, Germany

**Keywords:** Plant signalling, Drought

## Abstract

Plants have developed sophisticated mechanisms to survive in dynamic environments. Plants can communicate via volatile organic compounds (VOCs) to warn neighboring plants of threats. In most cases, VOCs act as positive regulators of plant defense. However, the communication and role of volatiles in response to drought stress are poorly understood. Here, we showed that tea plants release numerous VOCs. Among them, methyl salicylate (MeSA), benzyl alcohol, and phenethyl alcohol markedly increased under drought stress. Interestingly, further experiments revealed that drought-induced MeSA lowered the abscisic acid (ABA) content in neighboring plants by reducing 9-cis-epoxycarotenoid dioxygenase (*NCED*) gene expression, resulting in inhibition of stomatal closure and ultimately decreasing early drought tolerance in neighboring plants. Exogenous application of ABA reduced the wilting of tea plants caused by MeSA exposure. Exposure of *Nicotiana benthamiana* to MeSA also led to severe wilting, indicating that the ability of drought-induced MeSA to reduce early drought tolerance in neighboring plants may be conserved in other plant species. Taken together, these results provide evidence that drought-induced volatiles can reduce early drought tolerance in neighboring plants and lay a novel theoretical foundation for optimizing plant density and spacing.

## Introduction

Plants require light, water, and soil nutrients to grow. Unlike animals, however, plants cannot hunt or escape danger, and they need pollinators for reproduction. Many studies have shown that plants can perceive and respond to environmental signals and alter their physiology and morphology accordingly^1^. For example, when plants grow under high densities, shade-intolerant plants adapt to low light by reducing their branch numbers^2^. For reproduction, plants communicate with other organisms to disperse seeds. Plants also attract predatory and parasitic insects to protect themselves from herbivorous pests and microbes or to obtain resistance to disease and herbivores^3^. These capabilities ultimately allow plants to expand and protect themselves.

Plants produce and emit volatile organic compounds (VOCs) that serve many functions during growth and development. Plant–plant communication via herbivore-induced VOCs has been extensively studied^4^. Evidence for pathogen-induced VOCs has been found in many plant species, such as *Arabidopsis*^5^, maize^6^, and wheat^7^. These VOCs can also mediate disease resistance in neighboring plants. Many studies of plant–plant communication via VOCs have reported evidence of communication among neighboring plants that provides resistance to insects^8^. For example, plants growing near damaged neighboring plants become more resistant to herbivores than do those growing farther away, although most of the underlying associated physiological and genetic mechanisms remain unknown. Plants also respond to signals produced upon encountering abiotic stress, such as those produced in response to UV-C irradiation and salinity^9^. In the case of salt stress, bean plants respond to airborne cues from salt-stressed neighboring plants through an increase in their salinity tolerance^10^. Moreover, unstressed *Pisum sativum* plants are able to share the root system with drought-stressed neighboring plants by eavesdropping for stress cues at the root level^11^.

Therefore, we assume that there is a positive regulatory role of volatiles during plant defense. However, some studies suggest there is specific intent: if the exchange of information among plants can deter herbivores, both neighboring plants and emitters benefit^12^. This is a win-win situation rather than an act of absolute goodness.

The tea plant is one of the most economically important crop species in China, Japan, India, and Kenya. Tea plants synthesize, accumulate, and emit many volatile compounds, which play important roles in tea quality and plant performance^13,14^. Cold and drought are major environmental factors that affect the natural geographic distribution of plants^15^. In the wintering period of tea-growing areas in northern China, cold and drought are the two main stresses to tea plants^16^. Our previous studies showed that some volatiles emitted from cold-stressed tea plants play key role(s) in priming cold tolerance in neighboring plants^17^. Water is essential for tea plant growth and development. Therefore, drought-stressed plants improve the hydraulic redistribution of neighboring plants via changes to the root system^11^. However, it is unknown whether VOCs released from aboveground tissues participate in plant–plant communication under drought stress. In this study, we found that drought-induced VOCs from tea plants promote leaf wilting in neighboring plants. Methyl salicylate (MeSA) was found to play a key role in plant–plant interactions under early drought stress by inhibiting abscisic acid (ABA) biosynthesis in neighboring plants.

## Results

### Drought-stressed tea plants reduce the early drought tolerance of their neighbors

**Fig. 1:**
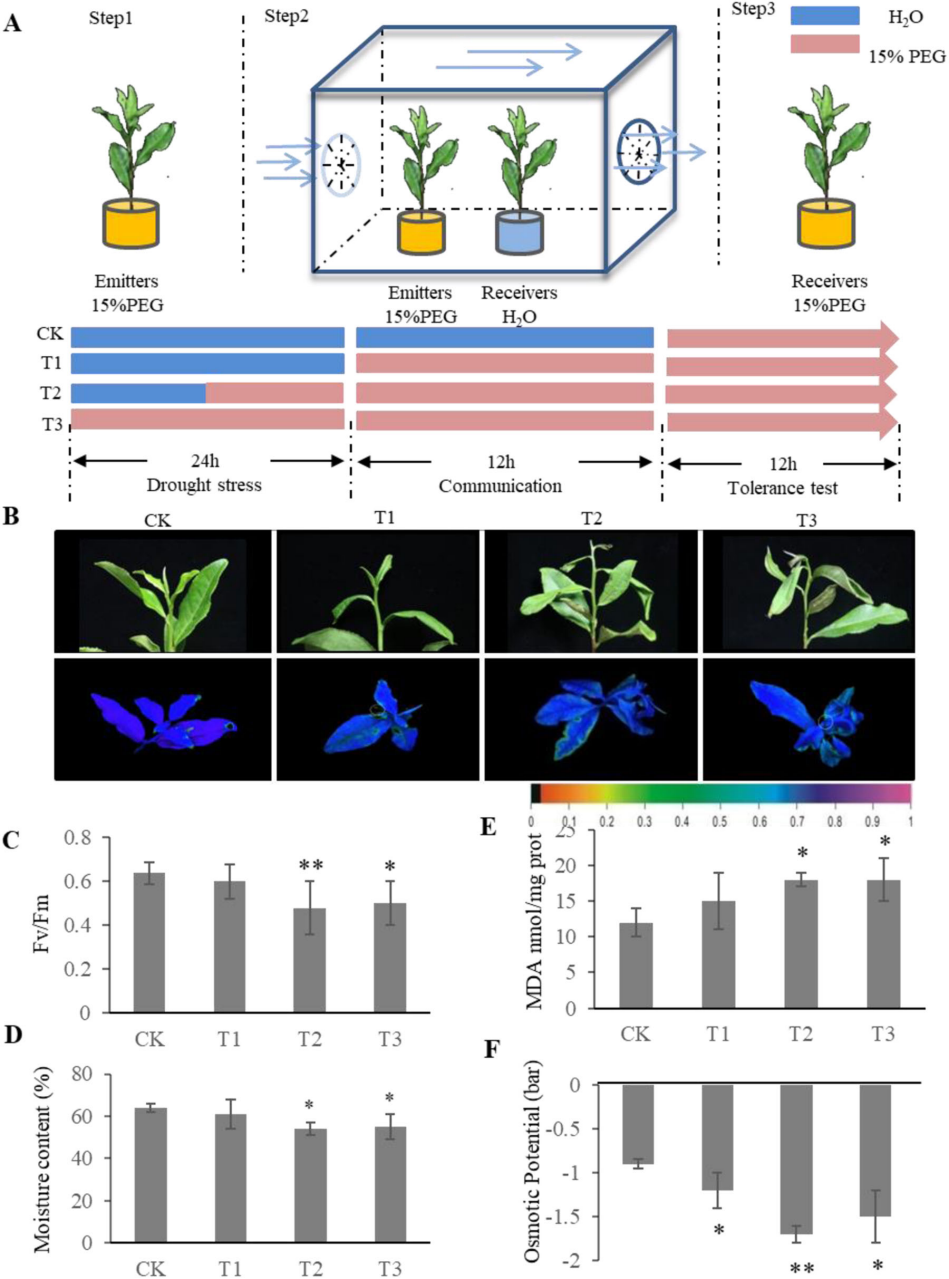
Experimental design and physiological analysis of drought-stressed receivers. **A** Communication experiment workflow. **B** Top row, degree of wilting of drought-stressed receivers in three different treatments (T1–T3) and the controls (CK); bottom row, chlorophyll fluorescence images of drought-stressed receivers in T1–T3 and the controls (CK). The color scale at the bottom of B ranges from 0 (black) to 1.0 (purple) and indicates the magnitude of the fluorescence signal. **C**–**F** *Fv*/*Fm* ratio, relative water content, MDA content, and osmotic potential of drought-stressed receivers in T1–T3 and the controls (CK). The data were presented as the means ± standard deviations (SDs) of six biological replicates. The asterisks indicate significant differences relative to the controls (Tukey’s HSD: *P < 0.05; **P < 0.01)

To test whether neighboring plants can interact with drought-stressed tea plants via a volatile exchange, plant–plant communication experiments were designed, which involved three steps (Fig. 1A). First, tea plants were treated with 15% polyethylene glycol (PEG) 6000 (emitters) for 0 h (T1), 12 h (T2), and 24 h (T3) (Step 1). Second, unstressed tea plants (receivers) communicated with the drought-stressed emitters for an additional 12 h under open-flow conditions, while emitters remained in PEG (Step 2). Receivers communicating with unstressed emitters served as controls (CK). Third, drought stress tolerance in the receivers was assessed (Step 3).

Surprisingly, the wilting degree of T1, T2, and T3 receivers that had communicated with drought-stressed emitters was more severe than that of the CK (Fig. 1B), suggesting that the volatiles released from the drought-stressed plants acted as regulators of plant defense. To confirm this phenomenon, the relative water content (RWC), chlorophyll fluorescence, osmotic potential, and malondialdehyde (MDA) content in T1, T2, and T3 receivers were measured and compared to those of the CK receivers. The RWC of the T2 and T3 receivers was significantly lower than that of the CK receivers (Fig. 1D), while there was no difference between the T1 and CK receivers. This was consistent with morphological phenotypes (Fig. 1B), where the T2 and T3 receivers showed more severe wilting than T1 the receivers did. Furthermore, chlorophyll fluorescence, an indicator of the first stage of leaf wilting, in the CK remained higher than that in the T2 and T3 plants (Fig. 1B, C).

Osmotic potential in plants decreases in response to drought stress^18^. Accordingly, the osmotic potential of the T2 and T3 receivers was dramatically lower than that of the CK (Fig. 1F). Lipid peroxidation was also measured via the MDA content. Similarly, the MDA content in the T2 and T3 receivers was higher than that in the CK receivers (Fig. 1E). These results indicated that drought-induced VOCs dramatically decreased early drought tolerance in the receivers.

**Fig. 2:**
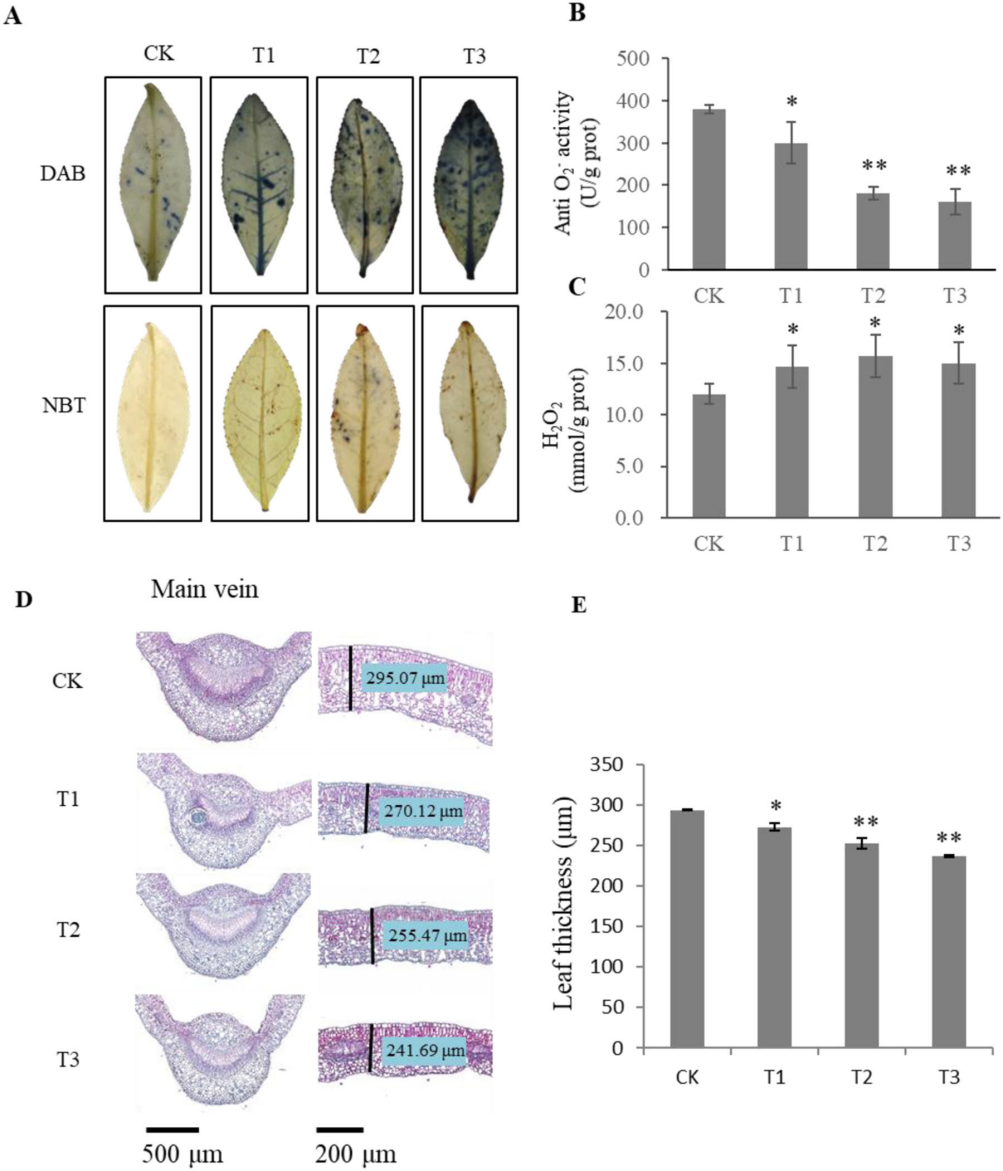
ROS content and microscopy observations of drought-stressed receivers. **A** Accumulation of H_2_O_2_ and O_2_^−^ as revealed by histochemical staining with DAB or NBT, respectively. **B**, **C** Quantitative measurements of O_2_^−^ (indicated by anti-O_2_^−^ activity, which is negatively proportional to the O_2_^−^ level) and H_2_O_2_. **D** Microscopy observations of cross-sections of paraffin-embedded leaves of drought-stressed receivers from T1–T3 and controls (CK); lignin is dyed red by safranin, and cellulose is dyed blue by fast green. The left side shows the main vein of the leaf; the right side shows the thickness of the leaf blade. **E** Measured leaf thickness. The data were presented as the means ± SDs of at least three measurements. The asterisks indicate significant differences relative to the controls (Tukey’s HSD: *P < 0.05; **P < 0.01)

Drought induces the accumulation of reactive oxygen species (ROS), including superoxide, hydrogen peroxide, and hydroxyl radicals^19^. In this study, two major ROS, H_2_O_2_ and O_2_^−^, were assessed in receivers based on 3,3′-diaminobenzidine (DAB) and nitro blue tetrazolium (NBT) histochemical staining, respectively. All T1, T2, and T3 receivers displayed more extensive DAB staining than did the CK receivers (Fig. 2A). This higher ROS accumulation in the T1, T2, and T3 receivers was also confirmed by quantitative measurements of H_2_O_2_ and O_2_^−^ (Fig. 2B, C).

Based on our microscopy observations of cross-sections, the leaves of the receivers were significantly thinner than those of the control plants (Fig. 2D, E). Compared with the CK plants, the T1, T2, and T3 receivers had more cellulose (blue parts stained by fast green) but less lignin in the xylem (red parts stained by safranin) (Fig. 2D), indicating that the observed changes in lignin could be associated with altered drought tolerance of tea plants.

These results indicated that, after drought treatments, compared with the CK receives, the receivers, especially T2 and T3, had a lower water content, a lower osmotic potential, higher MDA levels, and a higher ROS content. Taken together, our results demonstrated that receivers exposed to drought-induced VOCs were more susceptible to early drought stress than were CK receivers.

### VOCs emitted from drought-stressed tea plants

To determine which VOCs affected receivers, volatiles were collected from healthy tea seedlings and plants under different degrees of drought stress. Like in the communication experiment above, emitters were treated with 15% PEG 6000 for 0, 12, 24, and 48 h. VOCs released from drought-stressed tea plants were captured by needle trap microextraction (NTME) at the end of drought treatments and analyzed by gas chromatography-mass spectrometry (GC-MS).

**Fig. 3:**
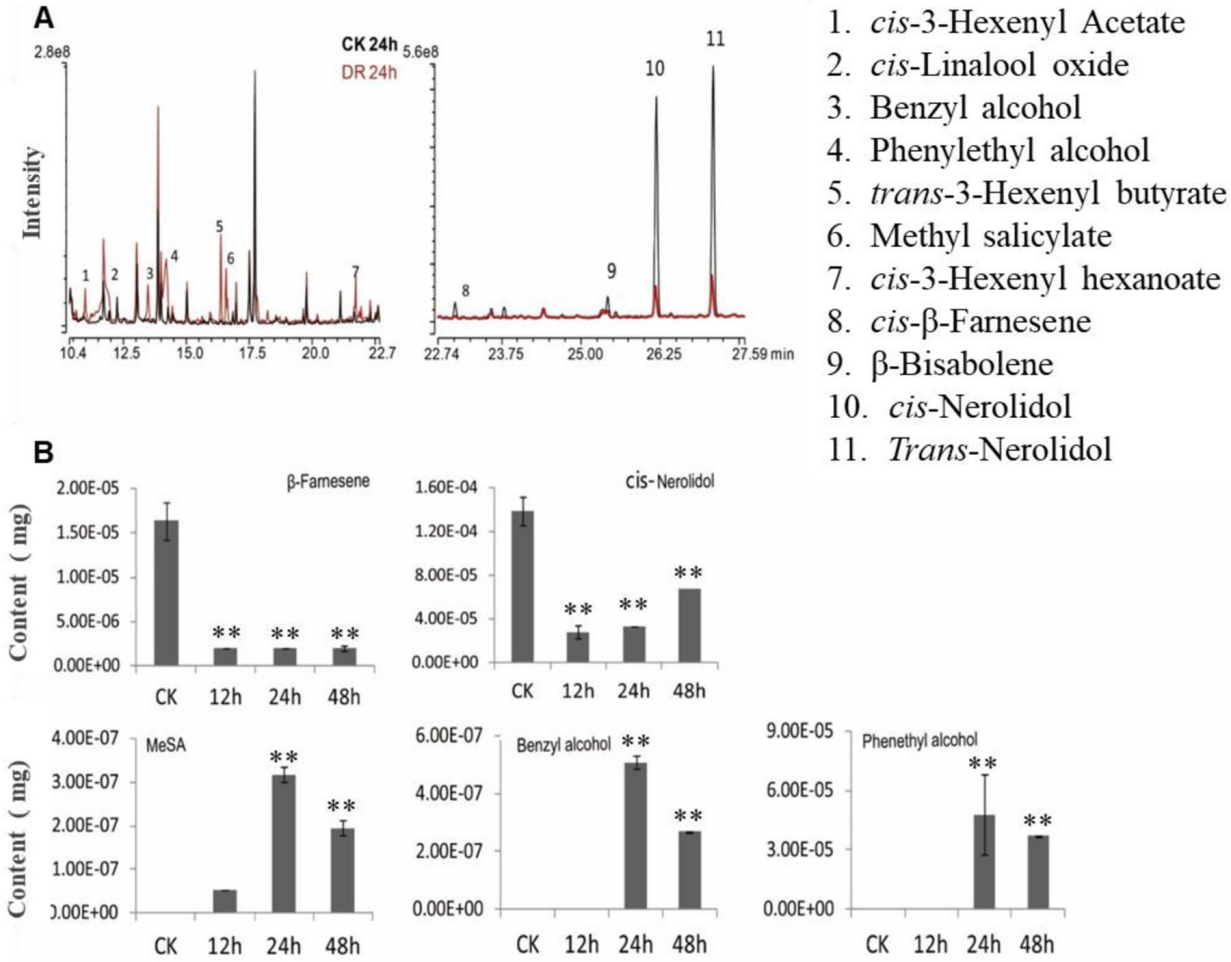
GC-MS analysis of volatile compounds under drought stress. **A** Volatile compounds under drought stress (DR24 h), as determined by GC-MS. **B** Quantitative analysis of β-farnesene, nerolidol, MeSA, benzyl alcohol, and phenethyl alcohol in plants under different drought stress treatments. The data were presented as the means ± SDs of six biological replicates. The asterisks indicate significant differences relative to the controls (Tukey’s HSD: *P < 0.05; **P < 0.01)

Based on the results that the drought tolerance of plants was reduced after they communicated with receiver plants via VOCs, we focused on volatiles that increased during drought stress. Three compounds, MeSA, benzyl alcohol, and phenethyl alcohol, exhibited significant increases in content (Fig. 3) during drought stress treatments. Quantitative analyses revealed that MeSA, benzyl alcohol, and phenethyl alcohol reached their maximum levels at 24 h (Fig. 3).

### Airborne MeSA reduces the early drought tolerance of neighboring plants

To investigate which compounds from emitters primed drought sensitivity in neighboring plants, exogenous volatile exposure experiments were performed. Tea plants were exposed to various concentrations of MeSA, benzyl alcohol, and phenethyl alcohol in 5-L sealed glass jars for 12 h; then, the drought tolerance of these plants with and without VOC exposure was compared. For all three compounds, the concentrations in the low (L) treatments were equal to the amounts released by plants in the drought stress treatments; the concentrations in the high (H) treatments were 100 times higher than those in the L treatments.

**Fig. 4:**
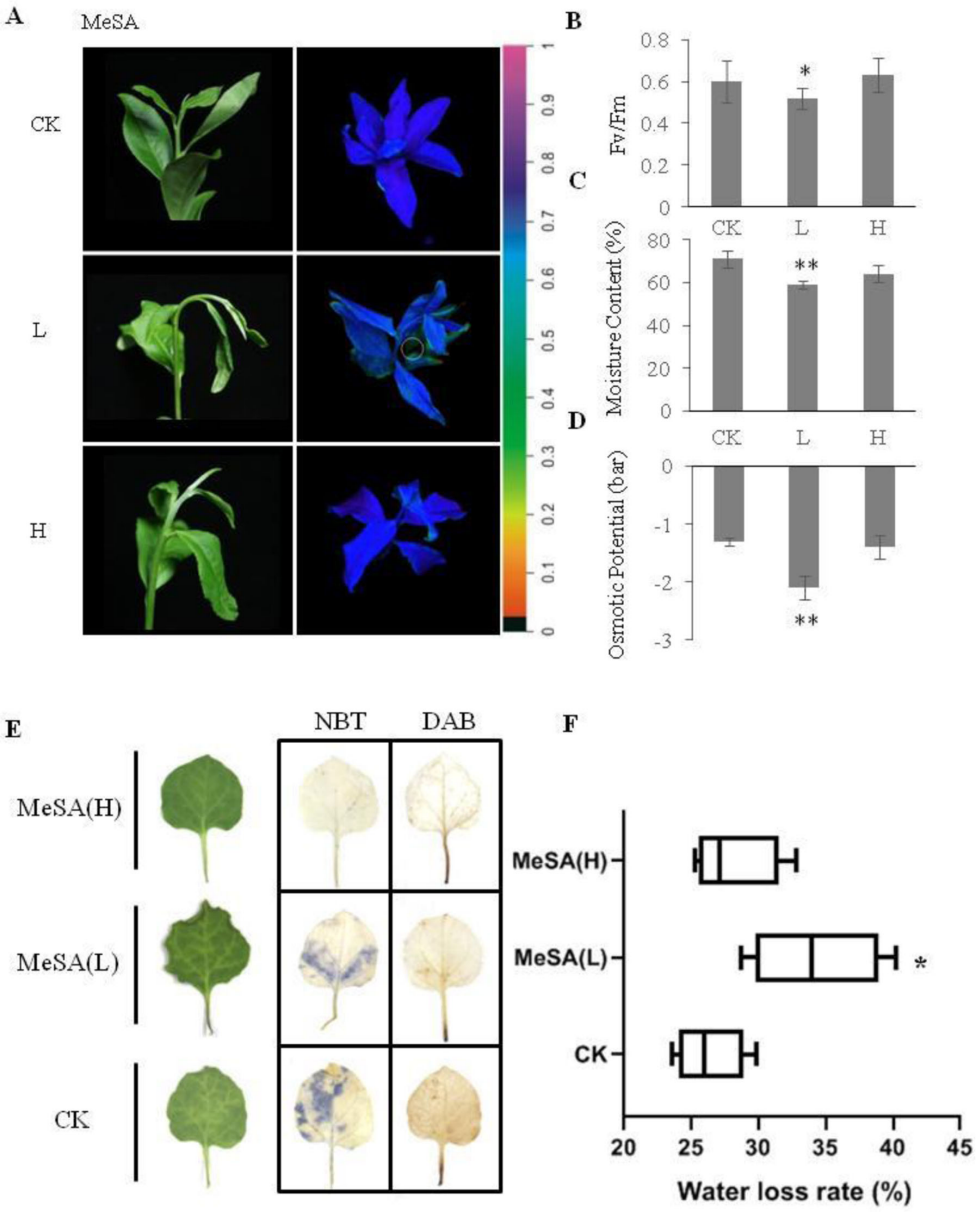
Phenotypes of tea seedlings and tobacco leaves exposed to MeSA. **A** Degree of wilting (left panel) and chlorophyll fluorescence images (right panel) of tea seedlings exposed to different concentrations of MeSA followed by drought stress. **B**–**D** Fv/Fm ratio, leaf water content, and osmotic potential of tea plants. **E** Wilting degree (left panel) and NBT and DAB staining images (right panel) of tobacco leaves exposed to different concentrations of MeSA followed by drought stress. **F** Water loss rate of MeSA-exposed tobacco leaves. The data were presented as the means ± SDs of six measurements. The asterisks indicate significant differences relative to the controls (Tukey’s HSD: *P < 0.05; **P < 0.01)

Interestingly, early drought tolerance was reduced in tea plants that were exposed to low concentrations of MeSA (L), which exhibited more severe wilting under drought conditions compared with that of plants in the other treatments (Fig. 4A). Similarly, chlorophyll fluorescence (Fig. 4B), leaf moisture content (Fig. 4C), and osmotic potential (Fig. 4D) in MeSA (L)-exposed tea seedlings decreased compared to those in the CKs. MeSA-exposed *Nicotiana benthamiana* also exhibited a similar severe wilting phenotype (Fig. 4E, F). Furthermore, NBT and DAB staining (Fig. 4E) showed that both MeSA (L) and MeSA (H) exposure reduced ROS. These results were consistent with our initial observations of healthy tea seedlings becoming more sensitive to drought after air exchange with drought-stressed tea seedlings, indicating that airborne MeSA played a key role in plant–plant communication of tea plants under early drought stress.

### Airborne MeSA decreases the ABA content of neighboring plants

To examine the mechanism underlying these observed effects, differentially expressed genes (DEGs) were compared between T2 receivers and CKs from the transcriptomic analysis. According to Kyoto Encyclopedia of Genes and Genomes (KEGG) enrichment analysis, the DEGs were mainly enriched in “plant hormone signal transduction” pathways (Supplemental Figs. 1, 2), suggesting that plant hormones play key roles in VOC-mediated plant–plant communication under early drought stress.

Plants produce and accumulate ABA under drought stress, which induces stomatal closure to avoid transpiration^20^. In plant hormone signal transduction pathways, there were key changes in the expression of proteins involved in ABA biosynthesis and signal transduction, including PYR (pyrabactin resistance)/PYL (PYR1-like), protein phosphatase 2 C (PP2C), and SNF1-related protein kinases (SnRKs), as well as in ABF transcription factors involved in the regulation of plant responses to abiotic stress (Supplemental Fig. 3). The expression of the 9-*cis*-epoxycarotenoid dioxygenase (*NCED*) gene (Supplemental Fig. 3), which encodes the rate-limiting enzyme of ABA synthesis, cleaves 9-*cis* xanthophylls to xanthoxin, a precursor of ABA and xanthoxin dehydrogenase (*ABA2*). ABA can be stored as glucose ester in tea seedlings^21^. The expression of abscisate of the beta-glucosyltransferase (*AOG*) gene also increased (Supplemental Fig. 3), the product of which regulates ABA conversion to its glucose ester, an inactive storage form of ABA. These results suggested that ABA biosynthesis and accumulation in neighboring plants were affected by drought-induced airborne cues, thus reducing early drought resistance.

**Fig. 5:**
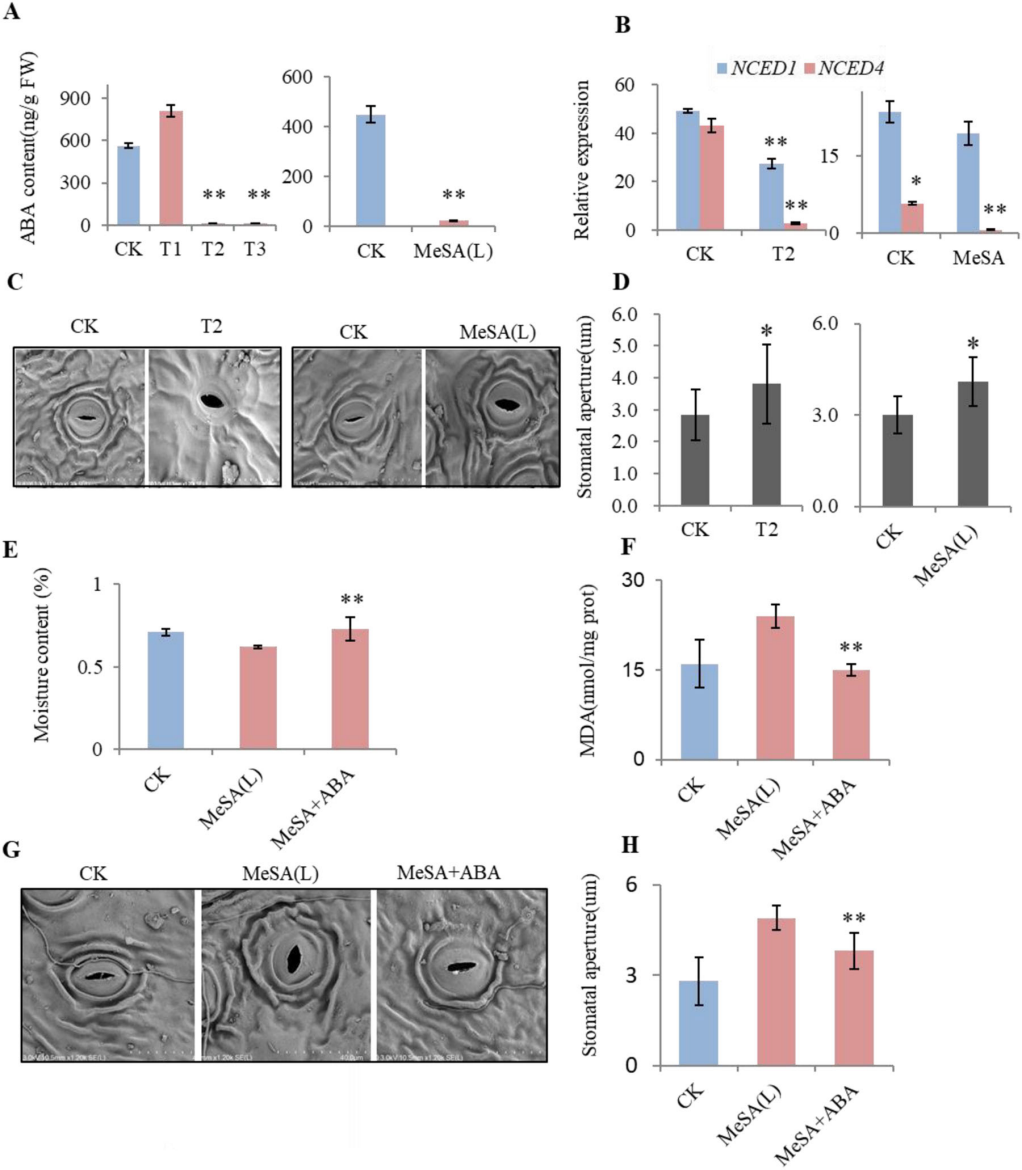
ABA content, relative gene expression, and stomatal aperture of tea seedlings exposed to MeSA. **A** ABA content of T1–T3 receivers and MeSA (L)-treated tea plants. **B** Expression levels of *NECD1* and *NCED4* in T2 and MeSA (L) tea plants. **C**, **D** Stomatal aperture of T2 and MeSA (L)-treated plants. **E**, **F** Leaf water content and MDA content of MeSA (L) and MeSA + ABA plants. **G**, **H** Stomatal aperture measurements of MeSA (L)-treated and MeSA + ABA plants. The data were presented as the means ± SDs of at least triplicate measurements. The asterisks indicate significant differences relative to the controls (Tukey’s HSD: *P < 0.05; **P < 0.01)

To determine the underlying mechanism of drought-induced MeSA, which made the receivers more susceptible to drought stress, we quantified the ABA content in tea plants with and without MeSA exposure. The ABA content in the T2 receivers was significantly lower than that in the CKs and T1 receivers, and ABA content in the MeSA-exposed tea plants was significantly lower than that in the CKs (Fig. 5A). Furthermore, the expression of *NCED1* and *NCED4* was significantly downregulated (Fig. 5B). In addition, the stomatal aperture in T2 receivers and MeSA (L)-exposed tea plants was larger than those in the CKs (Fig. 5C, D). Hence, it appeared that drought-stressed tea plants reduced early competition from neighboring plants via MeSA by inhibiting ABA biosynthesis in neighboring plants.

### MeSA-based induction of drought sensitivity in tea plants was restored by exogenous ABA

To determine whether the drought sensitivity of neighboring tea plants after MeSA exposure was caused by a reduction in ABA concentration, a 20 μM ABA solution was sprayed onto tea plants before exposure to MeSA (L) (MeSA + ABA). Tea plants sprayed with water were used as controls (CK). Compared with the CKs, the MeSA + ABA tea seedlings were more vigorous, with higher moisture content (Fig. 5E) and lower MDA content (Fig. 5F). Additionally, the stomatal aperture in MeSA + ABA tea plants was significantly smaller than that in the MeSA-exposed plants without exogenous ABA (Fig. 5G, H). Taken together, these results indicated that the MeSA-induced early drought insensitivity of tea plants could be rescued by the application of exogenous ABA.

Taken together, our results suggested that airborne MeSA could reduce early drought tolerance by decreasing the ABA content in neighboring plants, indicating crosstalk occurs between MeSA and ABA under drought stress.

## Discussion

Plants perceive and respond to environmental signals by changing their physiology and morphology^1^. However, plants rely on other organisms to help disperse seeds, protect against herbivorous insects and microbes, and resist disease and herbivores^3^. These interactions help sustain plant populations.

In a previous study of VOC-mediated communication between tea plants, induction of the green leaf volatile (Z)−3-hexenol from insects affected insect resistance in neighboring tea seedlings by increasing the content of (Z)−3-hexenol glycosides^14^. In addition to the volatiles typically produced by leaves, volatile terpenes such as α-farnesene, β-ocimene, and nerolidol are often released when a tea plant is attacked by insect pests; α-farnesene and β-ocimene can improve the resistance of neighboring tea plants to insect pests^22^. Tea seedlings that are subjected to cold stress release nerolidol, which enhances the antioxidant capacity of adjacent tea seedlings to improve their cold resistance^17^. These studies indicate that tea plants respond to VOCs induced in adjacent tea plants. However, the potential roles of VOCs during abiotic stress, such as drought stress, have not been characterized.

Here, we showed that after drought treatment, receivers, especially in the T2 and T3 experiments, exhibited decreased moisture content, decreased osmotic potential, increased MDA levels, and increased ROS contents (Fig. 2). Drought stress can alter tissue structure and anatomical traits. In our study, we showed that the leaves of the receivers were thinner than were those of control plants (Fig. 2D, E). Drought can also lead to changes in the composition of the cell wall and altered amounts of lignin^23^. The lignin content under drought stress can increase or decrease depending on the species^24,25^. Here, we showed that, compared with the control plants, the receivers accumulated more cellulose but less lignin in the xylem (Fig. 2D), indicating that these changes in lignin could be associated with altered drought tolerance in tea plants. Overall, these findings imply that drought-induced VOCs can reduce early drought tolerance in neighboring plants.

In tea plants, benzyl and phenethyl alcohols are stored as glycoconjugates in the form of β-glucoside and/or β-primeveroside^26^, and MeSA occurs as β-glucoside and/or glucose ester^27^. MeSA, benzyl alcohol, and phenethyl alcohol contents significantly increased after drought stress (Fig. 3), likely due to the disintegration of plant cells. Glycosides are released into the cytoplasm and hydrolyzed, resulting in the liberation of volatiles^28^.

Salicylic acid (SA), a metabolite of MeSA, plays an important role in the defense response of plants to pathogen attack^29^. Several studies also support a key role of SA in modulating plant responses to abiotic stresses^30,31^ such as chilling injury^32^, salt stress^33^, drought stress^34^, and heat stress^35^. In maize, pretreatment with SA was shown to induce the expression of antioxidant enzymes, which increased chilling tolerance^31^. Apart from modulating plant responses, excessive SA accumulation can induce programmed cell death, leading to a hypersensitive response to ozone (O_3_)^36^. Interestingly, we showed that drought-induced MeSA made neighboring plants more susceptible to drought stress (Figs. 1, 4). This effect of MeSA is reminiscent of that of cytokines and auxin, in which stomatal opening is promoted when the hormones are at low physiological concentrations but inhibited when they are at high concentrations^37,38^.

Plants produce and accumulate ABA under drought stress. ABA serves as a chemical messenger that induces stomatal closure through secondary messengers to avoid transpiration^20^, and ABA-deficient mutants are susceptible to drought stress^39^. PYR/PYL, PP2C phosphatases, SnRKs, and ABF transcription factors are involved in plant tolerance to abiotic stresses. For example, overexpression of PYL genes was shown to enhance drought tolerance^40^, whereas loss-of-function of SnRK or ABF transcription factor genes lead to reduced drought tolerance^41,42^. Here, we showed that low concentrations of drought-induced MeSA decreased the ABA content in neighboring plants, which inhibited stomatal closure. This mechanism appeared to be conserved in tobacco, although further research is needed. Overall, we provide the first evidence that drought-induced volatile cues can reduce early drought tolerance in neighboring plants.

## Methods

### Plant materials

One-year-old tea (*Camellia sinensis* var. *sinensis* cv. Shuchazao) plants were obtained from the Dechang Seedling Company. The roots were washed with deionized water, after which the plants were placed into plastic pots filled with nutrient solution according to Zhang et al.^43^. The tea plants were grown in a growth chamber at a temperature of 22 ± 1 °C, an irradiance of 270 μM m^−2^ s^−1^, and relative humidity of 45–50% under a 12-h photoperiod for at least 1 week. *Nicotiana benthamiana* plants were grown in pots in a growth chamber under a 16-h photoperiod at a 20/25 °C night/day temperature.

### Chemicals and reagents

PEG 6000, MeSA (≥98%), benzyl alcohol (≥99%), phenethyl alcohol (≥99%), β-farnesene (≥95%), nerolidol (≥98%), DAB, NBT, and ABA ( ≥ 98%) were obtained from Sigma (Shanghai, China).

### Drought stress treatments

To simulate drought stress and measured VOCs released from drought-stressed tea plants, the nutrient solution for tea plants was replaced with 15% PEG 6000 (w/v) according to Zhang et al.^43^ for 0, 12, and 24 h, after which they were then sealed in a 5-L glass case for 12 h. Tea plants were grown in a nutrient solution without PEG 6000 as controls. Volatiles were captured by NTME at the end of drought treatments and analyzed by GC-MS. After drought stress treatments, one bud and two leaves from the tea plants were collected for further experiments.

### Plant–plant communication experiments

To assess whether tea plants respond to drought-stressed neighboring plants, we designed a plant–plant communication experiment (Fig. 1A). First, six tea plants were treated with 15% PEG 6000 (emitters) for 0 h (T1), 12 h (T2), and 24 h (T3). Then, the emitters were placed upwind of six unstressed neighboring plants (receivers) for 12 h in a chamber; the emitters remained in 15% PEG 6000. After communication, the receivers were cultured in 15% PEG for 12 h to assess their drought stress tolerance. Receivers that had communicated with unstressed emitters were used as controls (CK). We assessed at least six biological replicates.

### Physiological measurements

To determine leaf RWC, the third leaf from the plants in each treatment was collected, immediately weighed (fresh weight), fixed at 120 °C for 20 min, and subsequently, oven-dried for 24 h at 80 °C (dry weight), according to the methods of Biman Kumar Dutta^44^. The maximum efficiency of photosystem II photochemistry (*F*v/*F*m) was measured by using a pulse-modulated fluorimeter Imaging-PAM (Walz, Effeltrich, Germany) after 30 min of dark adaptation. *Fv*/*Fm* was quantified using Imaging WinGegE; low *Fv*/*Fm* ratios indicate damage^45^. For osmotic potential, the third leaf of all seedlings was sampled (excluding the main vein). Tissue homogenates were collected by centrifugation and analyzed by vapor pressure osmometry (VAPRO 5600; Wescor Inc., Logan, American). At least three biological replicates were assessed for each measurement. The osmotic potential was calculated according to the following formula:

Ψs (bar) = −Ci × *R* × *T* × 10^−3^, where Ci is the instrument reading (mmol/kg), *R* is the gas constant (0.008314 MPa·L//mol/K), and *T* is the temperature (298.15 K).

Histochemical staining of leaves with H_2_O_2_ and O_2_^−^ was performed by placing the tissue in fresh solutions of 1 mg/mL DAB or NBT in the dark for 8 h, followed by decolorization in 80% alcohol at 90 °C.

For analysis of the MDA, H_2_O_2_, and anti-O_2_^−^ capacity, 0.2 g of leaf tissue was homogenized in 2 mL of cold extraction buffer (0.1 M phosphate buffer; pH 7.0). After centrifugation at 8000 rpm for 10 min, the supernatant was analyzed to determine plant malondialdehyde (MDA) (colorimetric method) production, hydrogen peroxide production, and superoxide anion production via assay kits (Nanjing Jiancheng Bioengineering Institute, Nanjing, China).

### Microscopy observations

Longitudinal cross-sections were obtained from paraffin-embedded leaves and observed with a light microscope (Nikon 80i). Leaf thickness was calculated using ImageJ (National Institutes of Health; https://imagej.nih.gov/ij/). Each experiment involved at least six biological replicates.

### Detection of drought-induced VOCs

To detect volatile compounds induced by drought stress, tea seedlings were subjected to 12-, 24-, and 48-h drought stress and then placed in 5-L glass jars. Next, 1 L of gas was pumped through a fiber using gas collection equipment for needle trap microextraction (NTME) (PAS Technology, Deutschland GmbH). GC-MS analysis was performed using a GC column (DA-5, 60 m× 0.25 mm, 0.25 μm film thickness, Folsom, USA) in conjunction with the following temperature program: holding at 40 °C for 5 min, ramping to 180 °C at 2 °C/min, holding for 1 min, increasing to 240 °C at 2 °C/min, and then holding again for 3 min. The column effluent was ionized by electron impact ionization (80 eV), and mass scanning was performed from 50 to 600 *m*/*z*. Released volatiles from the receivers were quantified by comparing the signal in the total ion chromatogram with that of the calibration curve.

β-farnesene: y = 2E + 12x − 4E + 06 R² = 0.99

nerolidol: y = 1E + 12x − 5E + 06 R² = 0.9994

MeSA: y = 6E + 07x − 512998 R^2^ = 0.9896

benzyl alcohol: y = 1E + 14x − 2E + 06 R² = 0.9847

phenethyl alcohol: y = 1E + 12x − 2E + 06 R² = 0.9937

### Volatile exposure experiment

To test the effects of volatile compounds on tea plants, MeSA, benzyl alcohol, and phenethyl alcohol were pipetted separately onto 100 mg of cotton wool. To begin the experiment, six plants were exposed to volatile compounds in a 5-L glass vessel (23 cm in diameter and 40 cm tall) for 12 h with low and high concentrations of each compound. The low (L) concentration of each compound was selected based on the content released by tea plants that were treated with drought stress. The high (H) concentration was 100 times higher than the L concentration. The amounts of VOCs used were 3 × 10^−7^ mg for MeSA (L), 3 × 10^−5^ mg for MeSA (H), 5 × 10^−7^ mg for benzyl alcohol (L)/phenethyl alcohol (L), and 5 × 10^−5^ mg for benzyl alcohol (L)/phenethyl alcohol (L). Tea plants exposed to the same amounts of dimethyl sulfoxide (DMSO) were used as controls. The exposed tea plants were then treated with 15% PEG 6000 for 12 h to evaluate drought tolerance. For tobacco exposure experiments, six *N. benthamiana* plants were exposed to L and H concentrations of MeSA for 12 h in a 5-L glass jar, as described above. Tobacco plants were exposed to the same amounts of DMSO as were the controls. After exposure, tobacco leaves were collected for drought tolerance analysis.

### ABA detection

ABA was extracted and analyzed by high-performance liquid chromatography (HPLC) according to a previously described method^46^, with some modifications, as described below. Samples weighing 100 mg were ground and placed in 1 mL of ethyl acetate on a shaker for 30 min and then centrifuged at 3000 rpm for 10 min. The supernatant was dried in a Speed-Vac (Labconco Centrivap Concentrator, Kansas City, USA) and reconstituted in 200 ml of 100% methanol for HPLC. A Dionex Ultimate 3000 UHPLC system (Thermo Fisher Scientific) equipped with an autosampler was used. HPLC was performed on a reverse-phase C18 column (1.8 μm, 100 mm × 2.1 mm) at a solvent flow rate of 0.2 ml min^−1^. The column temperature was set as 40 °C, and the injected sample volume was 1 μl. The mobile phases were solvent A, which consisted of water mixed with 0.1% formic acid (v/v), and solvent B acetonitrile. The solvent gradient was as follows: 0–2 min, 2% B; 2–3 min, 10% B; 3–8 min, 12% B; 8–13 min, 14% B; 13–15 min, 40% B; 15–17 min, 80% B; 17–19 min, 80% B; 19–19.1 min 2% B; and 19.1–20 min, 2% B. Each experiment involved at least three biological replicates, and each biological replicate included at least three technical replicates.

### ABA application experiments

One-year-old tea plants were exposed to L concentrations of MeSA for 12 h as described above; then, the exposed plants were sprayed with 0.1 mM ABA dissolved in water (MeSA + ABA). Control tea plants were sprayed with water after exposure to MeSA (L). Each experiment involved at least six biological replicates.

### Stomatal aperture bioassays

Leaves from tea plants were collected and placed in a buffer solution (50 mM CaCl2, 10 mM KCl, and 10 mM MES-Tris; pH 6.15) for 4 h to fully open the stomata. Subsequently, the abaxial epidermis of the plants' leaves was placed onto a slide, and images were taken using a Zeiss LSM880 (Germany). Stomatal apertures and aperture width of each stomatal pore were measured using ImageJ. Approximately 30 stomatal pores from the middle region of the leaves were examined for each treatment.

### RNA isolation and cDNA library construction

Total RNA was isolated from leaves of *Camellia sinensis* var. *sinensis* cv. Shuchazao using RNAiso Mate for Plant Tissue (Takara, Dalian, China) and RNAiso Plus (Takara, Dalian, China) according to the manufacturer’s instructions. Next, cDNA was synthesized from total RNA by reverse transcription using PrimeScript RT Master Mix (Takara, Dalian, China).

### RNA-Seq, assembly, and functional annotation

cDNA fragments were purified with a QIAQuick PCR Purification Kit, end-repaired, and ligated to Illumina sequencing adapters. Fragments were selected by gel electrophoresis, amplified via PCR, and paired-end sequenced with an Illumina HiSeq^TM^ 2000 (Gene Denovo Biotechnology Co., Ltd., Guangzhou, China) instrument.

After sequencing, the raw reads were filtered by removing low-quality reads that comprised more than 50% low-quality (*Q* value ≤ 10) bases to obtain high-quality clean reads. The rRNA-removed reads were assembled de novo using Trinity (v2.1.1), and the length distribution of the assembled unigenes was obtained. Unigene expression was calculated and normalized to reads per kilobase per million reads (RPKM). To annotate the unigenes, BLASTX (v2.2.29 + ) was used to search the unigenes against the National Center for Biotechnology Information (NCBI) nonredundant protein (Nr), SwissProt, KEGG, and Clusters of Orthologous Groups (COG) databases, with an E-value threshold of 1e-5.

### Quantitative real-time PCR-based analysis of *NCED*

Real-time PCR was performed according to published protocols with gene-specific primers designed by Primer 3 online. *Glyceraldehyde-3-phosphate dehydrogenase* (*GAPDH*) was used as an internal reference gene, and the relative expression was calculated using the 2^–ΔCT^ method^47^. The following primers were used: *GAPDH* forward (TTGGCA TCGTTGAGGGTCT), *GAPDH* reverse (CAGTGGGAACACGGAAAGC), *NCED* forward (TCAGCTCGGGTCTACCATGA), and *NCED* reverse (GTCCGGTGACTTGGTTCCAT). All the reactions were carried out via a CFX96™ System (Bio-Rad, USA) using the following temperature program: 95 °C for 3 min, followed by 40 cycles of 95 °C for 10 s and 62 °C for 30 s.

### Data analysis

The data were analyzed using SPSS Statistics (20.0) (SPSS, Inc., Chicago, IL, USA) and presented as the means ± standard deviations (SDs) of at least three biological replicates. Significance was determined at *P* < 0.05 by analysis of variance (ANOVA) followed by Tukey’s HSD test.

## Supplementary information

Supplemental Figures

## Data Availability

All the data generated in this study are included in this published article and its supplementary information.
