# Correction: Amplification of early drought responses caused by volatile cues emitted from neighboring tea plants

**DOI:** 10.1038/s41438-021-00708-7

**Published:** 2021-12-10

**Authors:** Jieyang Jin, Mingyue Zhao, Ting Gao, Tingting Jing, Na Zhang, Jingming Wang, Xianchen Zhang, Jin Huang, Wilfried Schwab, Chuankui Song

**Affiliations:** 1 State Key Laboratory of Tea Plant Biology and Utilization, International Joint Laboratory on Tea Chemistry and Health Effects, Anhui Agricultural University, 230036 Hefei, Anhui P. R. China; 2 Biotechnology Institute, Chengdu Newsun Crop Science Co., Ltd, 610212 Chengdu, P. R. China; 3 Biotechnology of Natural Products, Technische Universität München, Liesel-Beckmann-Str. 1, 85354 Freising, Germany

**Keywords:** Plant signalling, Drought

Correction to: *Horticulture Research*

10.1038/s41438-021-00704-x, published online 15 November 2021

After the publication of this article^1^, the authors became aware that the word of “tea” was not mentioned in the title. To make the title clear and complete, the word ‘tea’ is added to the title. The correct version of title is shown below:

*Amplification of early drought responses caused by volatile cues emitted from neighboring tea plants*

The original article has been corrected.
