# Supplementary material for: Amplification of early drought responses caused by volatile cues emitted from neighboring tea plants
Source: Hortic Res. 2021 Nov 15;8:243. doi: 10.1038/s41438-021-00704-x (PMC8593122; doi:10.1038/s41438-021-00704-x)
Supplement: Supplementary file 1 — Supplemental Figures [file 41438_2021_704_MOESM1_ESM.pdf]

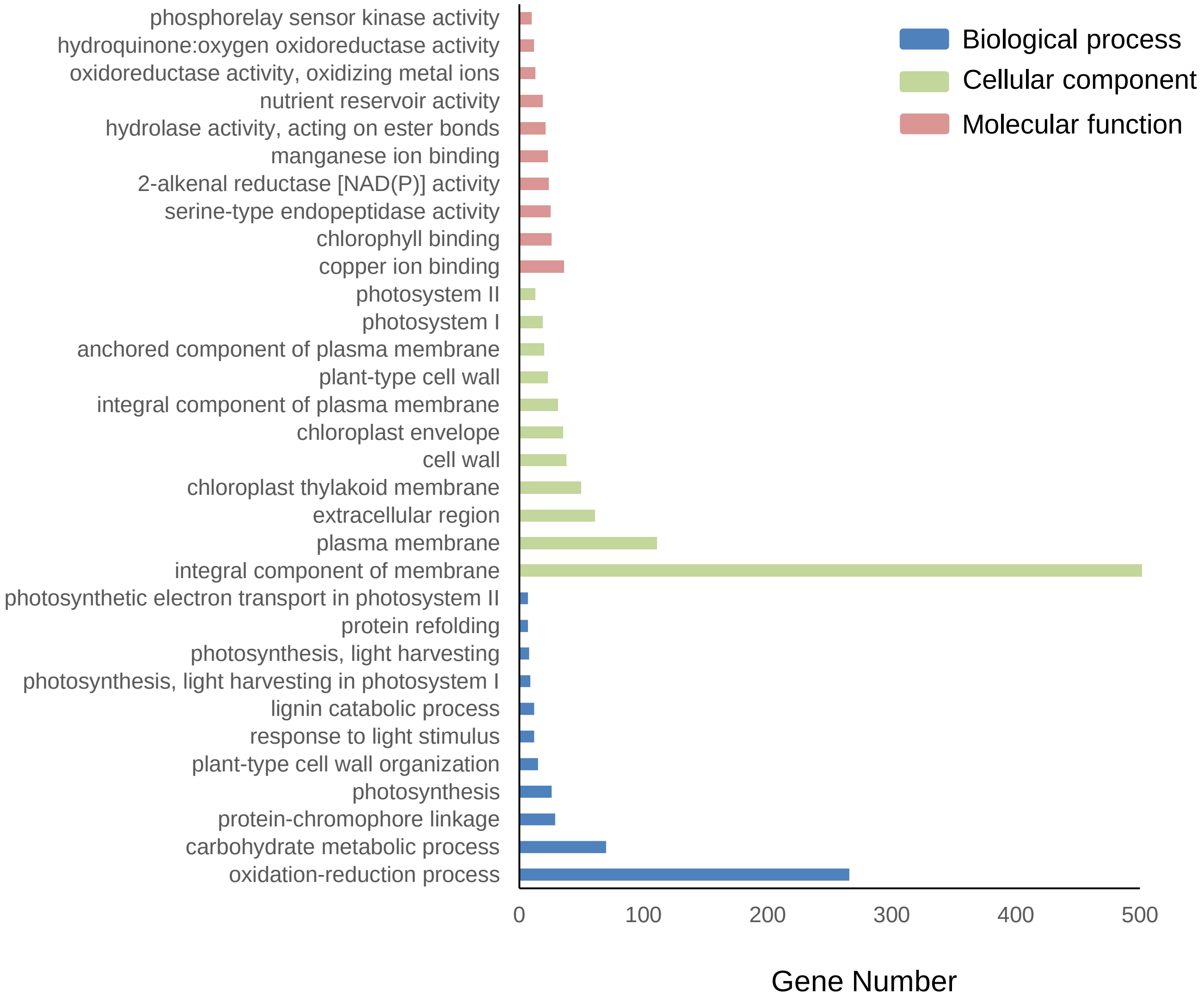

Supplemental Fig. 1

GO enrichment analysis of differentially expressed genes between control and T2 plants

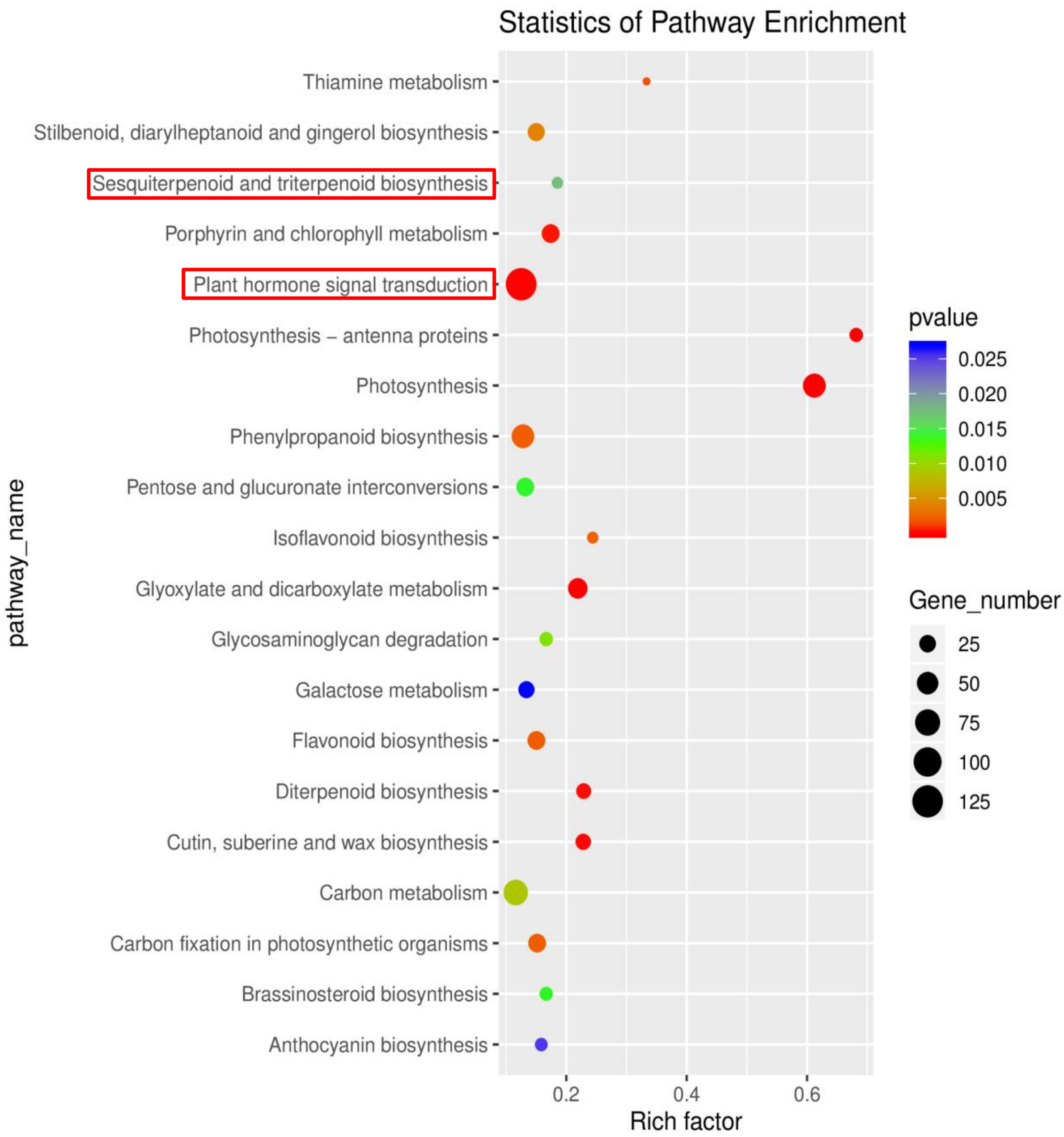

Supplemental Fig. 2

KEGG enrichment analysis of differentially expressed genes between control and T2 plants

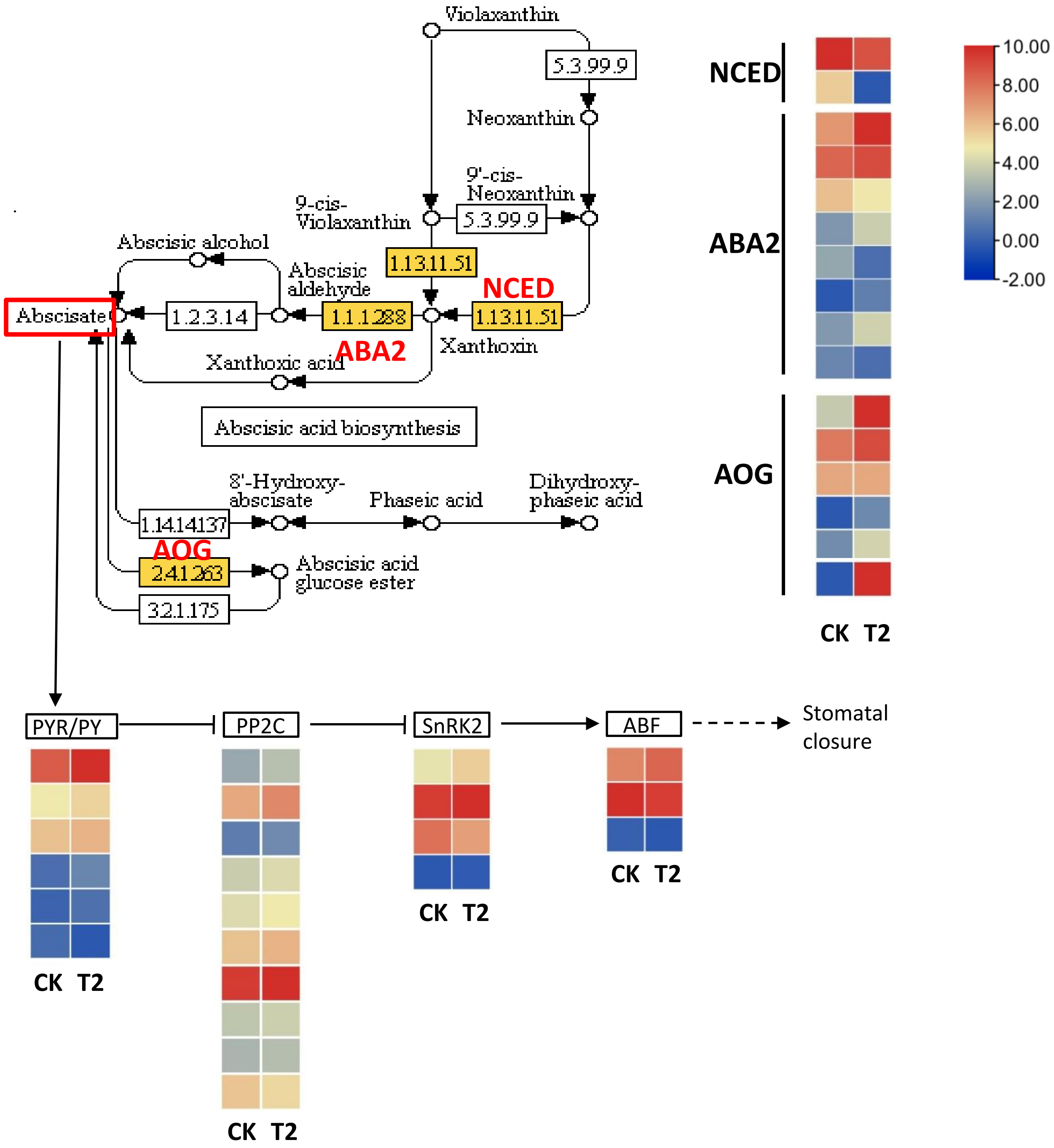

Supplemental Fig. 3

Heat map of changed genes in the ABA synthesis and transport pathway. *NCED,* 9-cis-epoxycarotenoid dioxygenase; *ABA2,* xanthoxin dehydrogenase; AOG, beta-glucosyltransferase; PYR, pyrabactin resistance; PYL (PYR1-like); PP2C, protein phosphatase 2C; SnRK2, SNF1-related protein kinases
